# Supplementary material for: Bringing legislation to life: Navigating intersectional barriers in implementing the Sindh Empowerment of Persons with Disabilities Act (2018) for the healthcare of children with disabilities
Source: PLOS Glob Public Health. 2026 Jun 4;6(6):e0006562. doi: 10.1371/journal.pgph.0006562 (PMC13235867; doi:10.1371/journal.pgph.0006562)
Supplement: S1 Text — (DOCX) [file pgph.0006562.s001.docx]

**Key Informant Interview Guide**

Challenges in Implementing Health-Related Provisions of the Sindh Empowerment of Persons with Disabilities Act 2018 for Children with Disabilities in Sindh, Pakistan

**Note to Interviewers**

This is a single semi-structured interview guide designed for use across all four categories of key informants involved in this study: (1) government officials and policymakers from the Department of Empowerment of Persons with Disabilities (DEPD), (2) district health administrators, (3) disability activists and non-governmental organization (NGO) representatives, and (4) pediatricians and healthcare providers.

Most questions are applicable to all stakeholders. Where a question is intended for specific stakeholder groups only, this is indicated immediately below the question in square brackets, for example: [For: Government officials and health administrators]. Probe questions are listed in italics beneath each main question and should be used flexibly based on the flow of conversation — not all probes need to be asked if the topic has already been addressed.

Interviews should be conducted in Urdu or Sindhi as preferred by the participant and are expected to last approximately 30–45 minutes. Audio recording should begin only after written informed consent has been obtained.

**Section A: Background and Role**

(Applicable to all stakeholders)

1. Could you briefly describe your current role and responsibilities, and how long you have been in this position?

- *What aspects of your work are most relevant to disability or health services in Sindh?*
- *Have you worked previously in any other role related to disability, child health, or social welfare?*

1. How familiar are you with the Sindh Empowerment of Persons with Disabilities (SEPD) Act 2018, particularly its health-related provisions?

- *Which provisions of the Act are most relevant to your role or work?*
- *Have you observed any changes in practice or policy since the Act came into force?*
- *Were you involved in any way in the development or consultation for this Act?*

**Section B: Governance, Leadership, and Intersectoral Coordination**

(Framework domain: Leadership and Governance)

**B.1 Coordination between DEPD and the Health Department**

1. How does the Department of Empowerment of Persons with Disabilities coordinate with the provincial Health Department to implement the health provisions of the SEPD Act?

*[For: Government officials and health administrators]*

- *Are there formal mechanisms for this coordination, such as joint committees, designated focal persons, or official correspondence?*
- *How often do such coordination activities take place?*
- *How are the health needs of children with disabilities specifically addressed within these coordination processes?*

1. From your perspective, how effectively do relevant government departments collaborate on disability-inclusive health services?

*[For: Disability activists and healthcare providers]*

- *Have you observed or experienced specific consequences of gaps in coordination between departments?*
- *What would effective collaboration between the health department and DEPD look like in practice?*

**B.2 Leadership Continuity and Policy Stability**

1. How does the frequent transfer or turnover of government officials affect the implementation of the SEPD Act, particularly its health-related provisions?

- *Can you describe a situation where a change in leadership disrupted a program or initiative for persons with disabilities?*
- *What mechanisms exist to preserve institutional knowledge and program continuity when officials are transferred?*
- *How does this affect child-specific services such as early intervention or referral pathways?*

**B.3 Private Sector Engagement**

1. The SEPD Act requires the private health sector to provide services to persons with disabilities. How has this provision been implemented in practice?

*[For: Government officials and health administrators]*

- *What incentives or accountability measures are in place to encourage private facilities to participate?*
- *Have any standard operating procedures or guidelines been shared with private providers regarding disability-inclusive care?*
- *What are the main obstacles to meaningful private sector engagement?*

1. In your experience, do private health facilities provide adequate or accessible services to children with disabilities?

*[For: Disability activists and healthcare providers]*

- *For what reasons do families of children with disabilities seek or avoid private health facilities?*
- *What financial barriers do families face when accessing private sector care?*
- *Are you aware of any private facility providing disability-inclusive services effectively?*

**Section C: Health Financing and Financial Protection**

(Framework domain: Health Financing)

**C.1 Budget Allocation**

1. What budget has been allocated by DEPD or the provincial government for implementing the health provisions of the SEPD Act?

*[For: Government officials and health administrators]*

- *Is there a dedicated budget line for disability-inclusive health at the provincial or district level?*
- *Are there specific funds allocated for the rehabilitation or healthcare needs of children with disabilities?*
- *How is health-related funding channelled — directly to DEPD, to district health authorities, or through other mechanisms?*

1. How do funding constraints at the system level affect the availability of health services for children with disabilities in your experience?

*[For: Disability activists and healthcare providers]*

- *Which services are most visibly affected by inadequate funding?*
- *Do families primarily rely on out-of-pocket payments, or are any government-funded services available to them?*

**C.2 Health Insurance**

1. The SEPD Act mandates the provision of affordable health insurance for persons with disabilities. What is the current status of this provision in Sindh?

- *Are there active plans or negotiations for a health insurance scheme that covers children with disabilities?*
- *How does the absence of structured insurance affect families financially?*
- *How does Sindh compare to other provinces — such as Punjab or Khyber Pakhtunkhwa — in terms of financial protection for persons with disabilities?*

*[Additionally, for government officials:]*

- *What specific challenges have been encountered in establishing an insurance mechanism?*
- *What is the expected timeline for a formal program to be operational?*

**C.3 Assistive Devices and Rehabilitation — Cost Burden**

1. How do families of children with disabilities finance assistive devices and rehabilitation services in Sindh?

- *Are any devices or rehabilitation services provided free of charge or subsidized through government programs?*
- *What do families typically spend out of pocket on items such as wheelchairs, hearing aids, or orthoses?*
- *How does children's rapid growth affect the ongoing financial burden of assistive devices?*

**Section D: Health Information Systems and Disability Data**

(Framework domain: Health Information Systems)

1. Does the provincial Health Information System currently capture data on patients with disabilities, including children?

- *Are standardized disability indicators included in routine health facility reporting?*
- *How is disability status recorded at the facility level — through clinical coding, functional assessment, or another method?*

*[For: Government officials and health administrators]*

- *Are there plans to integrate disability-disaggregated data into the Health Information System?*
- *What barriers exist to collecting and reporting disability data at health facilities?*

1. How is data on children with disabilities used to inform health planning and monitoring at the district or provincial level?

- *Can you give an example of how disability data has influenced a policy or program decision?*
- *What data gaps most limit effective planning for children with disabilities?*

1. How is the disability certificate used to link children with disabilities to health, rehabilitation, and social protection services?

- *How accessible is the certification process for families, particularly those in rural or remote areas?*
- *In your experience, what proportion of children with disabilities have obtained a disability certificate?*
- *What barriers prevent families from obtaining a certificate for their child?*

**Section E: Health Facility Infrastructure and Service Availability**

(Framework domain: Service Delivery — Health Facilities and Services)

**E.1 Physical Accessibility of Health Facilities**

1. How would you describe the physical accessibility of health facilities in your district for children with disabilities?

- *Do facilities have ramps, accessible seating, priority queuing arrangements, or equipment suitable for children with disabilities?*
- *Are sign language services or visual communication aids available at any facilities?*
- *What changes, if any, have been made to improve accessibility since the SEPD Act was enacted?*

1. Have you encountered situations where children with disabilities were unable to access a health facility due to physical barriers?

*[For: Disability activists and healthcare providers]*

- *Can you describe a specific example?*
- *How did the family or facility manage the situation?*

**E.2 Newborn Screening and Early Identification**

1. What newborn screening protocols currently exist in your facility or district for early identification of impairments or disabilities?

*[For: Health administrators and healthcare providers]*

- *Are standardized guidelines in place, for example those aligned with the Sindh Newborn Screening Act 2013?*
- *What happens when a newborn is identified as having a potential impairment — what care pathway follows?*
- *Is there a system to ensure families follow up for assessment and intervention after a referral?*

1. How are developmental delays or disabilities identified in children who present to health facilities after the newborn period?

*[For: Healthcare providers]*

- *Are any structured developmental screening tools used routinely in your facility?*
- *How confident are you in identifying and diagnosing different types of disability in children?*
- *What referral pathway is followed once a disability is diagnosed?*

1. Based on your work with families, how many report that their child's disability was identified early through an organized screening or referral system?

*[For: Disability activists]*

- *What do families typically describe about the process of obtaining a diagnosis for their child?*
- *Where do most families first become aware that their child may have a disability?*

**Section F: Health Workforce and Provider Competency**

(Framework domain: Service Delivery — Health Workforce)

1. What training have healthcare providers in your district or facility received on managing patients with disabilities, particularly children?

- *Is disability-inclusive care addressed in undergraduate or postgraduate medical or nursing curricula?*
- *Are there any in-service training programs or continuing medical education sessions on this topic?*

*[For: Government officials and health administrators]*

- *Has DEPD or the health department organized any capacity-building activities for healthcare providers on disability-inclusive care?*

1. How would you describe the knowledge, attitudes, and practices of healthcare providers toward children with disabilities?

- *Do you observe differences in the quality of care given to children with and without disabilities?*
- *Have you seen providers avoiding or delegating care for children with disabilities? What factors drive this?*
- *How do providers typically communicate with children who are non-verbal, deaf, or have intellectual disabilities — directly with the child, or primarily through caregivers?*

*[For: Disability activists — additionally ask:]*

- *What do families report about how they and their children are treated when they visit health facilities?*

1. What additional training or support would most help healthcare providers deliver appropriate care to children with disabilities?

*[For: Healthcare providers]*

- *Which communication skills are most lacking — for working with children who are autistic, deaf, non-verbal, or have physical disabilities?*
- *How should medical and nursing education be reformed to better prepare providers for disability-inclusive care?*

**Section G: Rehabilitation Services and Assistive Technology**

(Framework domain: Service Delivery — Rehabilitation and Assistive Technology)

1. What rehabilitation services are available to children with disabilities through public health facilities in Sindh?

- *Are physiotherapy, occupational therapy, speech therapy, or audiology services available at facilities or through a referral system?*
- *How do families typically access these services — through government facilities, NGOs, or private providers?*
- *What are the most significant gaps in rehabilitation service provision for children?*

1. How accessible and affordable are assistive devices for children with disabilities in your district?

- *Are devices such as wheelchairs, hearing aids, orthoses, or prosthetics available through any public facility or government program?*
- *What do families typically pay for assistive devices, and how does cost affect whether they access them?*
- *How does children's physical growth affect the need for updated or replacement devices, and how is this managed?*

1. Is there any organized government mechanism for providing free or subsidized corrective surgeries or medical procedures for children with disabilities?

- *How do families access surgical or specialized medical services when required?*
- *What role do NGOs or donor-funded programs play in filling this gap?*

**Section H: Overall Assessment and Recommendations**

(Applicable to all stakeholders)

1. In your view, what have been the most significant successes and failures in implementing the health provisions of the SEPD Act 2018 since it was enacted?

- *What factors have enabled any successes?*
- *What systemic factors have most constrained implementation?*
- *Are there provisions of the Act that remain entirely unimplemented?*

1. How do children with disabilities experience the health system differently from adults with disabilities, or from children without disabilities?

- *Are there specific healthcare needs of children with disabilities that are particularly neglected?*
- *What makes disability-inclusive care for children more complex or resource-intensive than for adults?*

1. What priority changes — in policy, financing, governance, or service delivery — would make the most meaningful difference for children with disabilities accessing healthcare in Sindh?

- *What can realistically be done in the near term, and what requires longer-term structural reform?*
- *What specific role should your department or organization play?*
- *Are there examples from other provinces in Pakistan or from other countries that Sindh should learn from?*

[End of interview — please stop the audio recording]
