# Supplementary material for: Bringing legislation to life: Navigating intersectional barriers in implementing the Sindh Empowerment of Persons with Disabilities Act (2018) for the healthcare of children with disabilities
Source: PLOS Glob Public Health. 2026 Jun 4;6(6):e0006562. doi: 10.1371/journal.pgph.0006562 (PMC13235867; doi:10.1371/journal.pgph.0006562)
Supplement: Attachment — Submitted filename: DETAILED PEER REVIEW REPORT Warukira Julius Njuguna.docx [file pgph.0006562.s002.docx]

**DETAILED PEER REVIEW REPORT**

**Manuscript Title:**
*Bringing Legislation to Life: Navigating Intersectional Barriers in Implementing the Sindh Empowerment of Persons with Disabilities Act (2018) for the Healthcare of Children with Disabilities*

**Journal:** *PLOS Global Public Health*
**Article Type:** Research Article
**Reviewer Role:** Warukira Julius Njuguna -Academic Reviewer

**1. Overall Evaluation and Contribution**

This manuscript addresses an **important and policy-relevant issue**: the gap between disability-inclusive legislation and its implementation within health systems, with a stated focus on **children with disabilities** in Sindh, Pakistan. The topic aligns well with *PLOS Global Public Health’s* mission to advance equity-oriented, implementation-focused public health research in low- and middle-income countries (LMICs).

The study contributes meaningfully by:

- Examining implementation barriers through a **health systems lens**
- Applying the **Missing Billion Disability-Inclusive Health System Framework**
- Engaging a **diverse group of stakeholders**, including policymakers, health administrators, clinicians, and disability advocates

However, while the study is **conceptually strong**, it currently suffers from **significant weaknesses in focus, clarity, methodological transparency, language precision, and journal compliance**, which limit its readiness for publication in its current form.

Overall, the manuscript demonstrates **high potential**, but it requires **major revisions** before it can be considered for publication.

**2. Major Comments (Critical Issues Requiring Substantive Revision)**

**2.1 Misalignment Between Stated Focus and Empirical Content (Children vs. General Disability)**

**Issue Identified:**
Although the title, abstract, and objectives emphasize *children with disabilities*, much of the empirical material including interview quotations, thematic analysis, and discussion addresses *persons with disabilities in general*, without consistently distinguishing child-specific experiences or health system needs.

Examples include:

- Quotes that refer broadly to “people with disabilities” without age differentiation
- Findings on governance, insurance, and assistive devices that are not explicitly child-centered
- Discussion sections that generalize adult disability challenges to children without analytical justification

**Why This Is a Serious Concern:**
This weakens internal coherence and may lead editors to question whether the study truly answers its stated research question. For a journal like PLOS GPH, conceptual precision is essential.

**How to Rectify:**

1. **Explicitly acknowledge this limitation early** in the Results section (not only in limitations).
2. Clearly state that:

Stakeholder perspectives often address system-wide disability issues

The analysis intentionally extracted **child-relevant implications** from broader narratives

3. Re-analyze findings to explicitly emphasize:

Child-specific service delivery (newborn screening, pediatric referral

systems)

Growth-related assistive technology needs

Caregiver dependency and decision-making

Pediatric rehabilitation gaps

4. Add a clearly labeled subsection in the Discussion:
   **“Child-Specific Implications of the SEPD Act”**

**2.2 Methodology: Insufficient Analytical Transparency**

**Issue Identified:**
The qualitative methodology is generally appropriate but **insufficiently detailed**, particularly regarding data analysis. Specific concerns include:

- Use of **Microsoft Excel** for qualitative analysis without justification
- Lack of clarity on how inductive and deductive approaches were combined
- Ambiguity regarding how the Missing Billion framework informed coding versus interpretation

**Why This Matters:**
PLOS places strong emphasis on **reproducibility and transparency**, even in qualitative research.

**How to Rectify:**

- Explicitly explain:

Whether Excel was used for **coding, data management, or matrix development**

Why Excel was appropriate given study scope and resources

- Add a step-by-step description of the analytical process:
  0. Initial open coding (inductive)
  1. Development of a preliminary codebook
  2. Mapping codes to the Missing Billion framework (deductive)
  3. Consensus-building between coders
- Clarify whether the framework:

Structured data collection

Structured analysis

Or was applied post-analysis

**2.3 Data Availability and PLOS Compliance (Critical Issue)**

**Issue Identified:**
The Data Availability Statement states: *“The data will be available upon request.”*
This **does not comply** with PLOS Global Public Health’s mandatory data-sharing policy unless a clear ethical justification is provided.

**Why This Is Critical:**
Non-compliance with data policies is a **common reason for desk rejection or delayed acceptance** at PLOS journals.

**How to Rectify:**
The authors must do **one** of the following:

1. Deposit **de-identified qualitative excerpts or coded datasets** in a public repository, **or**
2. Provide a detailed justification explaining:

Ethical risks (e.g., identifiability of officials)

Legal constraints

What data *can* be shared (e.g., codebooks, anonymized excerpts)

Who can be contacted for controlled access

This revision is **mandatory** for publication consideration.

**2.4 Ethical Reflexivity and Use of Appropriate Language**

**Issue Identified:**
The reflexive statement is valuable but contains **problematic terminology** and limited critical reflection.

Examples:

- Use of the phrase *“not disable”*
- Limited discussion of power relations between researchers and government actors

**Why This Matters:**
Language sensitivity is particularly important in disability research, and reflexivity is central to qualitative rigor.

**How to Rectify:**

- Replace all problematic language with **person-first terminology**
- Expand reflexive analysis to discuss:

Researchers’ institutional affiliations

Power asymmetries with participants

Potential influence on responses and interpretation

- Clarify how reflexivity informed analytic decisions

**2.5 Discussion Section: Length, Repetition, and Analytical Sharpness**

**Issue Identified:**
The Discussion section is **overly long**, with repeated arguments across governance, financing, HIS, and service delivery subsections. At times, it reiterates Results instead of interpreting them.

**Why This Matters:**
Strong discussions synthesize findings, rather than restating them.

**How to Rectify:**

- Reduce redundancy by:

Integrating governance and financing discussions

Highlighting cross-cutting barriers

- Focus on:

Interpretation

Policy implications

Transferability to other LMICs

- Explicitly link each recommendation to:

A specific study finding

A specific SEPD Act provision

**3. Section-Specific Technical and Editorial Comments**

**3.1 Title and Abstract**

- Title is strong but lengthy; consider tightening without losing meaning
- Abstract should:

Clarify the qualitative nature earlier

Explicitly state limitations regarding child-specific data

**3.2 Results Section**

- Some subsections would benefit from **clearer signposting**
- Ensure that each theme explicitly references:

How it affects children differently than adults

**3.3 Tables and Figures**

- Tables are informative but should:

Align with PLOS formatting standards

Include clearer captions explaining relevance to children

- Figures should be referenced more explicitly in the text

**3.4 Language, Grammar, and Style (Pervasive Issue)**

**Observed Problems:**

- Grammatical errors
- Awkward phrasing
- Inconsistent capitalization
- Inconsistent hyphenation (e.g., disability inclusive vs disability-inclusive)

**Recommendation:**
A **professional academic language edit** is strongly recommended prior to resubmission.

**3.5 References**

- Some references are outdated or peripheral
- Several URLs lack access dates or stable links
- Duplicate references noted

**Recommendation:**

- Update key global sources (WHO, UNICEF, World Bank)
- Ensure full compliance with PLOS reference style

**4. Strengths of the Manuscript**

Despite the above concerns, the manuscript has notable strengths:

- Strong policy relevance
- Rich qualitative insights
- Appropriate conceptual framework
- Multi-stakeholder perspective
- Clear contribution to disability-inclusive health systems research

**5. Final Recommendation to the Editor**

**Recommendation:** **MAJOR REVISION**

This manuscript has **strong potential for publication** in *PLOS Global Public Health*. However, substantial revisions are required to address:

- Child-specific analytical focus
- Methodological transparency
- Data availability compliance
- Language and editorial quality
- Analytical conciseness

Once these issues are adequately addressed, the manuscript would make a valuable contribution to the literature on disability-inclusive health policy implementation in LMICs.
