# Supplementary material for: Bringing legislation to life: Navigating intersectional barriers in implementing the Sindh Empowerment of Persons with Disabilities Act (2018) for the healthcare of children with disabilities
Source: PLOS Glob Public Health. 2026 Jun 4;6(6):e0006562. doi: 10.1371/journal.pgph.0006562 (PMC13235867; doi:10.1371/journal.pgph.0006562)
Supplement: Attachment — Submitted filename: Response to editorial comments.docx [file pgph.0006562.s004.docx]

PGPH-D-25-03724R1
Bringing Legislation to Life: Navigating Intersectional Barriers in Implementing the Sindh Empowerment of Persons with Disabilities Act (2018) for the Healthcare of Children with Disabilities
Dr Muhammad Asim

**Editorial Comment**

1. Please include a complete copy of PLOS’ questionnaire on inclusivity in global research in your revised manuscript. Our policy for research in this area aims to improve transparency in the reporting of research performed outside of researchers’ own country or community. The policy applies to researchers who have travelled to a different country to conduct research, research with Indigenous populations or their lands, and research on cultural artefacts. The questionnaire can also be requested at the journal’s discretion for any other submissions, even if these conditions are not met. Please find more information on the policy and a link to download a blank copy of the questionnaire here: <https://eur03.safelinks.protection.outlook.com/?url=https%3A%2F%2Fjournals.plos.org%2Fglobalpublichealth%2Fs%2Fbest-practices-in-research-reporting&data=05%7C02%7Casim.muhammad%40aku.edu%7C11b62f8c263a486b6e0508de78398144%7Ca5d4252a02f94e6096f09733baae4919%7C0%7C0%7C639080385577102932%7CUnknown%7CTWFpbGZsb3d8eyJFbXB0eU1hcGkiOnRydWUsIlYiOiIwLjAuMDAwMCIsIlAiOiJXaW4zMiIsIkFOIjoiTWFpbCIsIldUIjoyfQ%3D%3D%7C0%7C%7C%7C&sdata=sNFwl6wWxZjvkdNkgFuIQa4m3JAyQgmy7fzpSQcQsnA%3D&reserved=0>. Please upload a completed version of your questionnaire as Supporting Information when you resubmit your manuscript.

**Author’s comment: We have uploaded questionnaire on inclusivity in global research along with our article-on-article submission portal**

2. Please provide a detailed online Financial Disclosure statement. This is published with the article. It must therefore be completed in full sentences and contain the exact wording you wish to be published.

a) Please clarify all sources of financial support for your study. List the grants, grant numbers, and organizations that funded your study, including funding received from your institution. Please note that suppliers of material support, including research materials, should be recognized in the Acknowledgements section rather than in the Financial Disclosure.

b) State the initials, alongside each funding source, of each author to receive each grant. For example: “This work was supported by the National Institutes of Health (####### to AM; ###### to CJ) and the National Science Foundation (###### to AM).”

c) State what role the funders took in the study. If the funders had no role in your study, please state: “The funders had no role in study design, data collection and analysis, decision to publish, or preparation of the manuscript.”

For more information, please go to our submission guidelines:
<https://eur03.safelinks.protection.outlook.com/?url=https%3A%2F%2Fjournals.plos.org%2Fglobalpublichealth%2Fs%2Fsubmission-guidelines%23loc-financial-disclosure-statement&data=05%7C02%7Casim.muhammad%40aku.edu%7C11b62f8c263a486b6e0508de78398144%7Ca5d4252a02f94e6096f09733baae4919%7C0%7C0%7C639080385577163163%7CUnknown%7CTWFpbGZsb3d8eyJFbXB0eU1hcGkiOnRydWUsIlYiOiIwLjAuMDAwMCIsIlAiOiJXaW4zMiIsIkFOIjoiTWFpbCIsIldUIjoyfQ%3D%3D%7C0%7C%7C%7C&sdata=JgnSObLtlsma3f3d2gP3vOJFyXAClaRbSXoHyPdGeSY%3D&reserved=0>

**Reviewer’s comment: We have already provided this in the manuscript. I am copying the statement over here as well. Funding: This work was supported by National Institute of Health (Health Research Institute), Islamabad, Pakistan. This grant was awarded to MA and Grant Reference Number RIG-22/R2-111/RDC/AKU. The funder had no role in study design, data collection and analysis, decision to publish, or preparation of the manuscript.**

3. Please ensure that the funders and grant numbers match between the Financial Disclosure field and the Funding Information tab in your submission form. Note that the funders must be provided in the same order in both places as well.

**Author’s comment: It is matched**


4. Please update your online Competing Interests statement. If you have no competing interests to declare, please state: “The authors have declared that no competing interests exist.”

**Author’s comment:** It is mentioned at the end of manuscript and before the references. I am copying hrere. **Competing interests:** The authors have declared that no competing interests exist.

5. We note that your Data Availability Statement is currently as follows: "The data will be available upon request"

Please confirm at this time whether or not your submission contains all raw data required to replicate the results of your study. Authors must share the “minimal data set” for their submission. PLOS defines the minimal data set to consist of the data required to replicate all study findings reported in the article, as well as related metadata and methods (<https://eur03.safelinks.protection.outlook.com/?url=https%3A%2F%2Fjournals.plos.org%2Fglobalpublichealth%2Fs%2Fdata-availability%23loc-minimal-data-set-definition&data=05%7C02%7Casim.muhammad%40aku.edu%7C11b62f8c263a486b6e0508de78398144%7Ca5d4252a02f94e6096f09733baae4919%7C0%7C0%7C639080385577190594%7CUnknown%7CTWFpbGZsb3d8eyJFbXB0eU1hcGkiOnRydWUsIlYiOiIwLjAuMDAwMCIsIlAiOiJXaW4zMiIsIkFOIjoiTWFpbCIsIldUIjoyfQ%3D%3D%7C0%7C%7C%7C&sdata=lhw3R2jxeqW%2BFEkPs%2Bm0ZDaDaGZnzytkkjrcDTLsixI%3D&reserved=0>).

For example, authors should submit the following data:

- The values behind the means, standard deviations and other measures reported;
- The values used to build graphs;
- The points extracted from images for analysis.

Authors do not need to submit their entire data set if only a portion of the data was used in the reported study.

If your submission does not contain these data, please either upload them as Supporting Information files or deposit them to a stable, public repository and provide us with the relevant URLs, DOIs, or accession numbers. For a list of recommended repositories, please see <https://eur03.safelinks.protection.outlook.com/?url=https%3A%2F%2Fjournals.plos.org%2Fglobalpublichealth%2Fs%2Frecommended-repositories&data=05%7C02%7Casim.muhammad%40aku.edu%7C11b62f8c263a486b6e0508de78398144%7Ca5d4252a02f94e6096f09733baae4919%7C0%7C0%7C639080385577214295%7CUnknown%7CTWFpbGZsb3d8eyJFbXB0eU1hcGkiOnRydWUsIlYiOiIwLjAuMDAwMCIsIlAiOiJXaW4zMiIsIkFOIjoiTWFpbCIsIldUIjoyfQ%3D%3D%7C0%7C%7C%7C&sdata=51aXVoROjcO%2FospcrR7BGNYAhzIdgm5Vu3DXjQRHYQ0%3D&reserved=0>.

If there are ethical or legal restrictions on sharing a de-identified data set, please explain them in detail (e.g., data contain potentially sensitive information, data are owned by a third-party organization, etc.) and who has imposed them (e.g., an ethics committee). Please also provide contact information for a data access committee, ethics committee, or other institutional body to which data requests may be sent. If data are owned by a third party, please indicate how others may request data access.

**Author’s comment: The data availability statement are below that is the part of manuscript**

**Data Availability:
The qualitative interview transcripts contain potentially identifiable information from a small number of participants, including officials from the Department of Empowerment of Persons with Disabilities (DEPD), disability advocates, and physicians (genetic pediatric specialists). For ethical and confidentiality reasons, the full transcripts cannot be made publicly available. De-identified data underlying the findings reported in this article may be made available upon request to the Principal Investigator, Dr. Muhammad Asim (asim.muhammad@aku.edu). Requests may also be directed to the Chair Ethics Committee, Aga Khan University, Karachi (**[**erc.pakistan@aku.edu**](mailto:erc.pakistan@aku.edu)**).**

6. Please provide separate main figure files in .tif or .eps format only and ensure that all files are under our size limit of 10MB.

For more information about how to convert your figure files please see our guidelines: <https://eur03.safelinks.protection.outlook.com/?url=https%3A%2F%2Fjournals.plos.org%2Fglobalpublichealth%2Fs%2Ffigures&data=05%7C02%7Casim.muhammad%40aku.edu%7C11b62f8c263a486b6e0508de78398144%7Ca5d4252a02f94e6096f09733baae4919%7C0%7C0%7C639080385577239113%7CUnknown%7CTWFpbGZsb3d8eyJFbXB0eU1hcGkiOnRydWUsIlYiOiIwLjAuMDAwMCIsIlAiOiJXaW4zMiIsIkFOIjoiTWFpbCIsIldUIjoyfQ%3D%3D%7C0%7C%7C%7C&sdata=fJg73vuCS%2FygmhNCy88xxd5mlXFVdOibR7Nt%2FRKJ85w%3D&reserved=0>


Your manuscript has been returned to your account. Please log on to PLOS Editorial Manager at <https://eur03.safelinks.protection.outlook.com/?url=https%3A%2F%2Fwww.editorialmanager.com%2Fpgph%2F&data=05%7C02%7Casim.muhammad%40aku.edu%7C11b62f8c263a486b6e0508de78398144%7Ca5d4252a02f94e6096f09733baae4919%7C0%7C0%7C639080385577263793%7CUnknown%7CTWFpbGZsb3d8eyJFbXB0eU1hcGkiOnRydWUsIlYiOiIwLjAuMDAwMCIsIlAiOiJXaW4zMiIsIkFOIjoiTWFpbCIsIldUIjoyfQ%3D%3D%7C0%7C%7C%7C&sdata=zpk9dob8rS891domI0Hpyjgwc%2FlSglXrG%2BX2nHGRQRc%3D&reserved=0> to access your manuscript.

**Author’s response: The file has been uploaded along with the article. Please review figure 01.**
